# Collagenous gastritis, a new spectrum of disease in pediatric patients: two case reports

**DOI:** 10.4076/1757-1626-2-7511

**Published:** 2009-06-10

**Authors:** David Suskind, Ghassan Wahbeh, Karen Murray, Dennis Christie, Raj P Kapur

**Affiliations:** 1 Departments of Pediatrics, Children's Hospital and Regional Medical CenterUniversity of Washington, Seattle, WAUSA; 2 Departments of Laboratories, Children's Hospital and Regional Medical CenterUniversity of Washington, Seattle, WAUSA

## Abstract

Collagenous gastritis is a rare gastrointestinal disorder characterized in pediatrics by abdominal pain and anemia. The literature divides collagenous gastritis into distinct pediatric-onset and adult-onset phenotypes. As opposed to pediatric form, the adult form is associated with collagenous colitis and presents clinically with voluminous non-bloody diarrhea. There are over 25 case reports of collagenous gastritis of which 10 are pediatric cases. We present two cases of pediatric onset collagenous gastritis: one with a classic pediatric presentation, the other with findings typical of adult-onset disease. This is the first report of the adult-onset phenotype collagenous gastritis in a pediatric patient.

## Introduction

Collagenous gastritis is a rare gastrointestinal disorder characterized in pediatrics by abdominal pain and severe anemia. Typically, the gastric mucosa appears inflamed or nodular and histology reveals an irregular thickened collagenous subepithelial band, entrapped dilated capillaries, and intraepithelial inflammatory cells. Although rare, the literature divides collagenous gastritis into distinct pediatric-onset and adult-onset phenotypes. As opposed to pediatric-onset collagenous gastritis, the adult-onset form is associated with collagenous colitis and presents clinically with voluminous non-bloody diarrhea. To date, the adult-onset phenotype has not been reported in a pediatric patient.

The etiology of collagenous gastritis is unclear. No etiology has been identified in the pediatric form. In adult-onset disease, autoimmune, infectious, and medication-induced causes have been considered possible triggers. There are over 25-case reports of collagenous gastritis reported worldwide of which 10 are pediatric cases [1-8]. We present two cases of pediatric onset collagenous gastritis: one with a classic pediatric presentation, the other with findings more typical of adult-onset disease, including collagenous colitis. The potential significance of the association between collagenous gastritis and collagenous colitis is discussed in the context of a literature review.

## Case presentation

### Case Report 1: Classic pediatric-onset presentation

A 9-year-old female presented with fatigue, decrease exercise tolerance, intermittent nonspecific abdominal pain and pallor. She had no diarrhea, constipation, vomiting or fever. She had been adopted from China at age 18 months. Prior to adoption, she had been treated for iron deficiency anemia with iron supplementation. Her history included a positive PPD without radiographic anomalies. There was no history of medication use including NSAID or herbal/over the counter medications. She had no known drug allergies. Her biologic family medical history was unknown. Her growth and development were age appropriate. She had obvious pallor but otherwise her physical exam was within normal limits, without lymphadenopathy, hepatosplenomegaly or musculoskeletal deformity. Laboratory work-up was notable for hemoglobin of 5.7 gm/dl with MCV of 48 fl, low iron saturation and low total iron level. Reticulocytes were increased and a stool sample was heme-occult positive. Other studies, including a complete blood count, metabolic panel including albumin, eosinophil count, inflammatory markers, *Helicobacter* antibodies and celiac screening, were normal. She underwent esophagogastroduodenoscopy for further evaluation of her anemia. Endoscopy revealed diffuse gastric nodularity with associated erythema. Biopsies revealed collagenous gastritis. She was initiated on iron therapy. Over the next four months, her fatigue and pallor improved, with complete resolution of her laboratory abnormalities. Since her symptoms abated, no other treatment was considered. Months after discontinuing iron, her MCV and iron indices were slightly abnormal, but she remained asymptomatic (without any abdominal pain). After iron was restarted, her labs again normalized. She remains asymptomatic with normal laboratory studies.

### Case Report 2: Adult-onset phenotypic presentation

A 15-year-old male with an early childhood history of food allergies developed nonbloody diarrhea. Initially, stools were loose, “mashed potato-to-watery” in consistency and occurred 3-5 times a day but increased to 10-15 stools per day with associated urgency and tenesmus over the first two months. After 2 months, he developed intermittent sharp left upper quadrant pain a few times per week and a large number of oral ulcers. He also had intermittent fever (up to 102°C), a 10 lb weight loss, and fatigue. He denied any joint pain, loss of appetite, or rash.

Laboratory evaluation after 3 months of symptoms including stool culture, ova and parasite, complete blood count, liver enzymes, and inflammatory markers were normal. Colonoscopy was grossly normal but histology showed subepithelial collagen deposition.

A few weeks later his fatigue and diarrhea worsened. His physical examination revealed splenomegaly and pallor. Laboratory tests showed pancytopenia (WBC, 2.9 K/mm^3^; absolute lymphocyte count, 551/mm^3^; absolute neutrophil count, 1450/mm^3^; platelets, 143 K/mm^3^; hematocrit, 24.7%) with an elevated sedimentation rate and C-reactive protein. He was transferred to our facility for further evaluation. Physical examination revealed pallor, a weight of 71.1 kg (50-75%), and a height of 182.1 cm (75-90%). His abdomen was soft and nondistended. The tip of his spleen was palpable just below the left costal margin without evidence of hepatomegaly, ascites or abdominal masses. His perianal exam was normal with no skin tags, or fistula. Stool was heme-occult negative.

Laboratory studies confirmed pancytopenia with elevated sedimentation rate and CRP, and revealed mild hepatitis (AST 106 units/L, ALT 66 units/L). Other findings included normal immunoglobulin levels (IgG, IgM, IgA and IgE), normal C3 complement, low C4 complement (10 mg/dL; normal 16-52), normal CH50 total haemolytic complement, and the following serological results: anticardiolipin IgM (23 MPL; normal < 15), ASCA IgA/IgG (negative), Anti OmpC (negative), pANCA (negative), Cbir1 (39.9 EU/ml; normal < 21), anti-gliadin IgA (12 U/mL; normal < 11) anti-gliadin IgG (99 U/mL; normal < 11). The infectious work-up including evaluation for mycoplasma pneumonia, parvovirus, respiratory syncytial virus, adenovirus, influenza A & B, parainfluenza, cytomegalovirus, Epstein-Barr virus and Herpes simplex virus 1&2 was negative. Bone marrow biopsy revealed hypercellular bone marrow with marked erythroid hyperplasia, megaloblastic changes, and a left shift in myeloid maturation with many bands consistent with peripheral consumption. Cytology was negative for malignant cells.

Diagnosis of autoimmune hemolytic anemia with pancytopenia secondary to splenic sequestration was made. Patient was begun on 65 mg of prednisone per day with normalization of his blood counts and resolution of his gastrointestinal symptoms.

**Figure 1.**
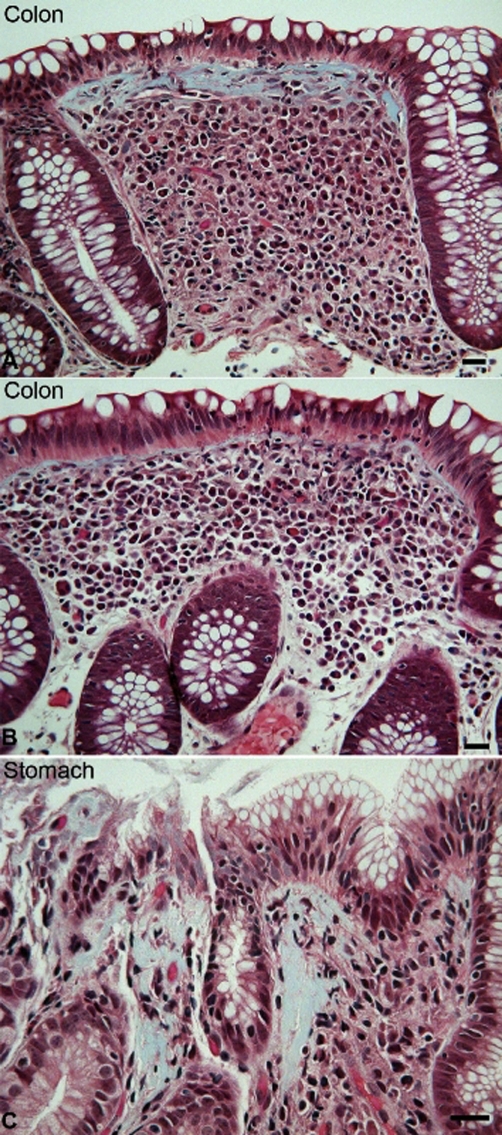
**(A)** A photomicrograph of a colonic biopsy from the patient shows a representative field of sub-epithelial collagen accumulation (blue), mild lymphocytic inflammation of the overlying epithelium, and diffuse chronic inflammation of the lamina propria. **(B)** An adjacent field in the same biopsy shows similar changes, except the sub-epithelial collagen table is not thickened. **(C)** Focal sub-epithelial collagen accumulation and associated inflammation is also evident in this field from the patient's gastric biopsy. Bars = 20 microns; Mallory trichrome stain.

The prednisone was slowly weaned over 5 months. One month after prednisone cessation, the patient again developed loose, non-bloody, non-mucousy stools (approx 2-3 times per day) associated with generalized abdominal pain. Endoscopy and repeat colonoscopy revealed normal appearing esophagus, colon and terminal ileum, mild patchy gastric erythema and moderate flattening, scalloping and erythema throughout the duodenum. Microscopic examination of mucosal biopsies showed normal esophageal histology, mild/moderate chronic active gastritis with patchy subepithelial collagen deposition (some areas > 30 microns), moderate chronic duodenitis with patchy villous blunting and mild subepithelial collagen accumulation, terminal ileum with patchy enteritis with lymphoid hyperplasia, and pan-colonic diffuse moderate chronic active colitis with foci of marked subepithelial collagen deposition (30-50 microns) (Figure 1). Tissue transglutaminase was normal. The pathology was consistent with collagenous gastritis and colitis and the patient was treated with lansoprazole and mesalamine. His symptoms resolved and he has remained asymptomatic on therapy for over a year.

## Discussion

Collagenous gastritis is an idiopathic disorder seen in both pediatric and adult patients. The disease phenotype is quite distinct in each population. In children, collagenous gastritis is characterized by severe anemia and abdominal pain. Typically, the stomach is nodular and inflamed with the colon not being affected. Adult onset collagenous gastritis is associated with collagenous colitis and presents with voluminous watery diarrhea. We present two cases of pediatric-onset collagenous gastritis: one with classic pediatric findings, the other with features typical of adult-onset disease, including associated collagenous colitis. This is the first reported case of the adult-onset phenotype seen in a pediatric patient.

In both forms of collagenous gastritis, the diagnosis is made by histology, which reveals distinctive findings. Gastric biopsies reveal a thick hyalinized collagenous band within the subepithelium of the gastric mucosa [9]. The collagenous bands are greater than 10 micrometers thick. The anemia found in the pediatric onset collagenous gastritis is believed to be due to hemorrhage from dilated capillaries entrapped in the abnormal collagenous matrix [3]. A similar histologic pattern is seen in adult collagenous gastritis but without associated anemia. The inflammatory cell response is predominantly a mononuclear infiltrate with few neutrophils and eosinophils in the lamina propria. The number of intraepithelial lymphocytes often exceeds 20 per 100 epithelial cells (normal 3-5 per 100 epithelial cells) [4]. In collagenous colitis associated with the adult form of collagenous gastritis, the subepithelial band of subepithelial collagen in the colonic mucosa varies from 7 to 100 micrometers, while normal is less than 7 micrometers [9].

Collagenous gastritis is rare. A total of 10 pediatric and 17 adult case reports have been described in the medical literature (Table 1). Lagorce-Pages et al. delineate two subsets of patients with collagenous gastritis: 1) collagenous gastritis occurring in children and young adults presenting with severe chronic anemia due to gastrointestinal bleeding, a nodular pattern on endoscopy, and a disease limited to the gastric mucosa without evidence of colonic involvement, and 2) collagenous gastritis associated with collagenous colitis occurring in adult patients presenting with chronic watery diarrhea [4]. In this context, our second patient expands the disease phenotype for collagenous gastritis in pediatric patients to include features previously only described in adults. To the best of our knowledge, he is the youngest individual reported with both collagenous gastritis and collagenous colitis.

**Table 1.** Clinico-endoscopic features of collagenous gastritis in paediatric patients

Case	Reference	Age/Sex	Presentation	Endoscopic findings	
				Stomach	Colon
1	Colletti and Trainer 1989 [2]	15/F	Epigastric pain, haematemesis	Nodularity	Normal
2	Cote et al. 1998 [3]	9/F	Severe anaemia	Erythema and erosions	Unknown
3	Lagorce-Pages et al. 2001 [4]	11/M	Severe anaemia	Nodularity	Normal
4	Meunier et al. 2001 [5]	11/NK	Severe anaemia	Macroscopic gastritis	Unknown
5	Meunier et al. 2001 [5]	12/NK	Severe anaemia	Macroscopic gastritis	Unknown
6	Park et al. 2005 [6]	11/M	Severe anaemia	Nodularity	Normal
7	Ravikumara, et al. 2007 [7]	9/F	Severe anaemia, epigastric pain	Nodularity	Normal
8	Kori et al. 2007 [8]	12/F	Nausea, vomiting and anemia	Thickened, nodular gastric body and fundus with exudate	Normal
9	Kori et al. 2007 [8]	12/F	Anemia and epigastric pain	Raised plaques and nodular gastric body and fundus	Normal
10	Kori et al. 2007 [8]	12/F	Epigastric pain	Nodular gastric body and antrum with erosions	Unknown
11	Present Case	9/F	Anemia and nonspecific abdominal pain	Diffuse gastric nodularity and erythema	Unknown
12	Present Case	15/M	Non bloody diarrhea	Patchy gastric erythema	Normal

The etiology of collagenous gastritis is unknown. Colletti et al., in a series of six children, reported an association with diabetes mellitus in one child, and psoriasis and achalasia in another. None of these findings were present in either of our patients. No other associations have been reported in pediatric patients. In adult-onset collagenous gastritis/colitis, autoimmune, infectious, and medication-induced (NSAIDs) causes have been considered possible triggers for the disease. In addition, it has been associated with putative immune-based disorders including celiac disease [10], inflammatory bowel disease [11], and a variety of systemic autoimmune diseases [12]. Our second patient had autoimmune hemolytic anemia, in addition to collagenous gastritis/colitis, which now must be added to this list of associated conditions. Collectively, these associations support the hypothesis that collagenous gastritis/colitis has an immune basis.

Although a genetic predisposition to adult phenotype collagenous gastritis has not been established, familial examples of collagenous colitis have been observed. Interestingly, different members of the same family may develop either lymphocytic or collagenous colitis supporting a similar underlying pathophysiology [1]. A link has also been suggested between collagenous colitis and inflammatory bowel disease [11], which is not the case for pediatric collagenous gastritis. In adult-onset collagenous gastritis, there appears to be no genders predominate in the medical literature; while in the pediatric literature there is a slight predominance of female patients, but so few pediatric examples have been published that the significance of this trend is unclear.

The natural history of the collagenous gastritis with or without colitis is incompletely elucidated. In adults, a chronic intermittent course characterizes the majority of patients [13], with no significant mortality risk or periods of severe deterioration. Furthermore, diarrhea may resolve with or without treatment, although relapses can occur. The pediatric cases reported thus far would indicate more recalcitrant disease. Winslow et al. describes the clinicopathologic evolution of collagenous gastritis in a single patient during a 12-year period. A hundred and nine biopsy specimens of gastric mucosa from 19 different endoscopic procedures where evaluated for severity and distribution of collagenous gastritis in a single patient. Relative to biopsy specimens from age and sex matched control subjects, the patient's biopsy specimens showed a significantly lower number of antral gastrin cells, along with a significant corpus endocrine cell hyperplasia, suggesting an increased risk of endocrine neoplasia. Gastric corpus biopsy specimens revealed an active, chronic gastritis, subepithelial collagen deposition, smooth muscle hyperplasia, and mild to moderate glandular atrophy [14]. Although, Winslow, et al. raises concerns about the potential for adenocarcinoma secondary to findings of intestinal metaplasia and reactive epithelial changes inderterminate for dysplasia, these concerns are speculative.

Various therapies have been trialled in the pediatric form of collagenous gastritis, including corticosteroids, ranitidine, misoprostil, sucralfate, 5-ASA and hypoallergenic diets, with little efficacy. The medical literature differs from our clinical experience, which suggests there is a spectrum of disease severity and responsiveness in children with collagenous gastritis. In the adult form of collagenous gastritis/colitis, therapies which resulted in improvement include cessation of NSAIDs, institution of a gluten-free diet (if associated with celiac disease), and loperamide. In addition, aminosalicylates, sulfasalazine, budesonide, cholestyramine and prednisone have been shown to be effective [15].

We present the first case of pediatric collagenous gastritis with collagenous colitis. When collagenous gastritis occurs in association with collagenous colitis, the clinical characteristics, activity, and associations mirror those in adults. Although rare, collagenous colitis should be considered in children with collagenous gastritis, particularly if they suffer from chronic diarrhea and/or autoimmune disorders.
